# Assessment of visual contrast sensitivity in biotoxin-exposed individuals using four testing methods

**DOI:** 10.1080/07853890.2026.2646821

**Published:** 2026-04-06

**Authors:** Ingrid Astrid Jimenez-Barbosa, Sean Di Lizio, Seong Beom Ahn, Benjamin Heng, Janet Kim, Amanda J. Watson, Sieu K. Khuu

**Affiliations:** a School of Optometry and Vision Science, The University of New South Wales, Sydney, Australia; b Digital Media Research Centre, School of Communication, QUT, Brisbane, Australia; c Mould Awareness Council, Brisbane, Australia; d Macquarie Medical School, Faculty of Medicine, Health and Human Sciences, Macquarie University, Sydney, Australia; e Integrative Medicine, N Plus Clinic, St Leonards, New South Wales, Australia; f PANDIS, Limited, Sydney, Australia

**Keywords:** Contrast sensitivity, neurotoxicity, chronic inflammatory response syndrome, biomarkers

## Abstract

**Background:**

Environmental biotoxins, such as mould, are linked to neurological and visual dysfunction, including impaired visual contrast sensitivity (VCS). Testing VCS alterations is recommended as a biomarker for biotoxicity. This study compared four VCS tests in detecting VCS deficits in individuals with clinical signs of biotoxicity.

**Methods:**

VCS was measured in 28 biotoxin-exposed individuals and 30 controls using four VCS tests: Shoemaker handheld chart, Online Contrast Sensitivity Test (OCST^TM^), Clinic CSF App, and an Experimental VCS test. Neurotoxicity symptoms were assessed with the modified Q16 questionnaire, and pupil size was measured under standard lighting.

**Results:**

Biotoxin-exposed participants had smaller pupil diameters (mean difference: 0.703 mm, *p* < 0.0001) and higher neurotoxicity scores (45.50 ± 13.0 vs. 22.00 ± 7.7). Contrast sensitivity was significantly reduced in exposed participants on digital VCS tests only. The Experimental VCS test demonstrated the highest diagnostic performance (100% sensitivity; 60–80% specificity), followed by OCST^TM^ and the Clinic CSF App. The Shoemaker handheld chart did not distinguish between groups.

**Conclusion:**

These results highlight the effectiveness of digital VCS testing and symptom questionnaires as practical tools for detecting visual and neurological impairments in individuals with suspected biotoxin exposure

## 1. Introduction

Biotoxicity, resulting from prolonged exposure to harmful biological toxins from mould and other environmental pollutants, has been linked to a range of neurological and systemic health conditions. These include Chronic Inflammatory Response Syndrome (CIRS), mycotoxicosis, and other immune-related disorders. Individuals affected by biotoxicity often report symptoms such as cognitive dysfunction, chronic fatigue, headaches, dizziness, and vision disorders [1]. Despite the growing recognition of these conditions, their diagnosis remains controversial due to symptoms that overlap with other disorders (e.g. allergies) and the limitations of conventional diagnostic tools.

Current screening methods for biotoxicity primarily rely on blood sample analyses to detect inflammatory markers, immune dysfunction, and toxin-specific antibodies. However, these tests are often time-consuming and costly and may not always provide conclusive results. Accordingly, there is a strong impetus for more cost-effective and reliable approaches and methodologies that can confirm and quantify the impact of biotoxins on health. Several approaches have assessed for changes to visual function as a sigh for neurotoxicity. It has been recognised that vision is a valid index for neurological processes, and its assessment provides a non-invasive means of assessing brain function and its change. Visual contrast sensitivity (VCS) testing is a well-established tool for assessing neurological function and has been employed as a potential tool for detecting signs of biotoxicity [2–4].

Typically, VCS corresponds to the minimum relative light intensity required for a stimulus to be detected from the background. Different methodologies present discrete targets, such as perimetric visual field technologies, optotypes (e.g. Pelli-Robson Chart), or traditional sinusoidal gratings [5]. The latter stimulus has clinical value as testing can be performed with grating of different spatial frequencies to measure the contrast sensitivity function (CSF), which provides a sensitivity profile or ‘window of visibility’ that characterises the minimum levels of contrast required to detect different levels of spatial detail [6]. The mechanisms underlying the contrast sensitivity function are also well understood and are characterised by narrow band pass spatial channels [7].

Measuring the CSF has been a mainstay in clinical vision, and its application to detect neurological change has long been recognised [4,8].

Previous studies have shown that VCS testing may play a crucial role in the early identification of impairments resulting from exposure to various chemicals and organic and inorganic neurotoxins such as organic solvents, pesticides and heavy metals [9–11].

Importantly, VCS impairments following exposure to these neurotoxins may share similar pathophysiological mechanisms with mould‑related biotoxicity. These mechanisms include oxidative stress, neuroinflammation, and disruption of cholinergic signalling in cortical and subcortical visual pathways [12–16]. The demonstrated sensitivity of VCS to such neurological insults supports its value as a non‑invasive window into central nervous system integrity, particularly where standard clinical imaging or electrophysiology may fail to reveal abnormalities and underscores the importance of VCS testing as an effective tool for facilitating timely interventions and implementing preventive measures to mitigate neurotoxic effects.

As mentioned, VCS has already been applied to detect signs of neurological change (manifesting as a reduction in VCS) from mould and other biotoxin exposures [17]. In particular, the Shoemaker handheld chart and a digital version of this test (Online Contrast Sensitivity Test, OCST^TM^) have been designed to provide a quick method of quantifying the CSF. These tests measure the ability to distinguish contrast at different spatial frequencies, which the neurotoxic effects of exposure to mould and other biotoxins can impair. The chart form of this test consisted of images of oriented gratings (vertical, tilted to the right or left) of progressively higher and lower contrast levels. The task requires judgement of the orientation of the gratings and the CSF derived from noting the contrast level needed to judge the orientation of the grating for each spatial frequency. However, this test has limitations, particularly the fact that the orientations, spatial frequencies, and contrast steps are fixed and therefore subjected to learning and practice effects and limited accuracy, respectively. Some of these limitations have been addressed in the computerised version of the OCST^TM^ in which gratings are presented randomly and sequentially; however, spatial frequency levels and contrast steps remain fixed, potentially limiting detection sensitivity. Additionally, accurate results require proper lighting, screen calibration, and at least 20/50 visual acuity.

Despite evidence that VCS testing is capable of detecting signs of neurological change from biotoxin and chemical exposure, a key challenge lies in the standardization and customization of assessments. Because there is no standard approach to measuring VCS, many methods have been reported in the literature that differ in stimuli, thresholding procedures, task, and the degree of customisation. Fundamental to these methods is the attempt to balance test accuracy for efficiency by varying the number of stimuli/spatial frequencies and or contrast steps. Testing at more spatial frequencies and finer contrast steps may provide greater sensitivity and characterisation of the CSF, but this is unnecessary (particularly for quick screening purposes) and time-consuming (and therefore not clinically suitable) as testing may involve more trials. On the other hand, testing efficiency can be improved by reducing the number of trials by presenting only a small number of stimuli and large contrast steps, but at the expense of reducing accuracy, as fewer trials and finer steps are not available to reach the actual threshold. Additionally, as mentioned, VCS can be influenced by various factors, including lighting conditions, screen resolution, and patient-specific variables. Therefore, test customisation may be essential for optimizing test effectiveness.

Given the importance of VCS testing in both clinical and research settings, continuous refinement of its methodologies is essential to enhance diagnostic reliability. The present study explored the validation of contrast sensitivity testing by comparing the capability and sensitivity of 4 different VCS tests in detecting biotoxicity-related visual impairments. All tests measured the CSF but applied different approaches and testing platforms, and importantly, vary in the degree of customisation. We evaluated the effectiveness of three commercialised VCS tests—the Shoemaker handheld chart, the OCST^TM^, and the Clinic CSF App 2.0.10. Along with a non-commercialised, research-focused Experimental VCS test, which we have used to characterise visual change due to neurotoxicity from organic solvents and pesticide exposure [10,11,14]. While commercial tests have limited customisation and standard proprietary thresholding approaches, the Experimental VCS test is highly customisable, allowing testing across many spatial frequency levels and adjustable finer contrast steps.

All VCS tests included in the present study have been used to report deficits in contrast sensitivity associated with biotoxin or chemical exposure. The study’s objective was to determine efficacy through comparison, particularly in the sensitivity of these tests detecting changes in contrast sensitivity, which would be crucial in monitoring treatment effectiveness and providing better patient outcomes. Through this comparison, we aimed to emphasize the need for enhancing these assessments to improve both their accuracy and clinical applicability in detecting neurotoxic impairments related to biotoxicity exposure. Additionally, comparative analyses of various test formats offer valuable insights into the strengths and limitations of existing tools, facilitating further improvements in testing accuracy and applicability.

In addition, the suitability of the modified Q16 neurotoxic symptoms questionnaire was explored. This questionnaire is a self-report screening tool designed to detect early signs of neurotoxicity, particularly in individuals exposed to neurotoxic substances like solvents and pesticides, among others. Previous research [10,18] has well established the validity and sensitivity of the modified Q16 neurotoxic symptoms questionnaire as a way of quantifying the symptoms of neurotoxicity, and in this study, it was applied to quantify common neurotoxic symptoms related to biotoxin exposure and associate the Q16 with VCS performance.

As an additional sign of neurotoxicity, pupil size measurements were also taken for all participants. Previous studies have reported significant alterations in pupil size from neurotoxicity (due to toxins such as pesticides and organic solvents) [14], and this measure may be another indication of possible autonomic nervous system change associated with exposure to biotoxins.

## 2. Materials and methods

### 2.1. Study population

This cross-sectional case-control study was part of a larger three-year research project examining the health impacts of biotoxin exposure. The component reported in this manuscript was conducted over one year, from January 2023 to December 2023. The study included 28 participants who had been recently diagnosed (within the previous 12 months) with biotoxin-related illness due to mould exposure, confirmed by a general practitioner specialised in this area. A control group of 30 non-exposed individuals was recruited from the same geographical area through community advertising and word-of-mouth referral. Controls were age‑matched to the exposed participants to minimise the potential confounding effects of age on contrast sensitivity. Data collection took place between February and August 2023. All assessments, including structured questionnaires, contrast sensitivity testing, and pupil size measurements, were conducted during the same session to ensure consistency across participants. Participants who required prescription glasses due to reduced near visual acuity wore them throughout all study procedures.

The sample size for this study was calculated using power analysis with PASS software, targeting for 80% statistical power and a significance level of 5%. The calculation was based on the difference in questionnaire scores between the exposed (mean = 48.0, SD = ±1.31) and non-exposed (mean = 21.65, SD = ±1.65) groups, as derived from previous applications of the modified neurotoxic symptoms Q16 questionnaire [10,14,18]. Note that the Q16 questionnaire does not measure visual contrast sensitivity directly; rather, it captures self-reported neurotoxic symptoms, including visual difficulties that may occur secondary to reduced contrast sensitivity. Accordingly, the sample size estimation was explicitly based on questionnaire‑derived symptom scores rather than CS outcomes.

Cohen’s d was calculated to estimate the effect size, resulting in a value of approximately 17.73. Using this effect size and the desired power, the required sample size was determined to be 28 participants for the exposed group and 30 participants for the non-exposed control group. The difference in group size reflected the output of the PASS power analysis, which generated the smallest number of participants needed in each group to achieve the target power based on the difference in questionnaire scores. Because the computation treats each group independently when determining the minimum sample size, the resulting estimates were not identical. Both values were used to ensure the study met the predefined power and significance criteria.

It included 28 participants recently diagnosed (within the past year) with biotoxin-related illness due to mould exposure, confirmed by a general practitioner specialised in this area. A control group of 30 non-exposed individuals with no history of exposure was also recruited. Data collection took place between February and August 2023 using structured questionnaires. All participants who required prescription glasses due to reduce near visual acuity wore them during the study procedures. Additionally, all clinical measurements were performed during the same session to ensure methodological consistency and reduce intra-participant variability.

The inclusion criteria for cases were clinically diagnosed biotoxin patients exposed to mould and with at least a medium-low level (score: 33–48) of neurotoxic symptoms as indicated by the modified Q16 neurotoxic symptoms questionnaire. The participants comprising the exposed group were aged between 27 to 60 years and confirmed (by a general practitioner with expertise in biotoxicity) to have a biotoxin-related illness due to mould exposure within the past year.

The non-exposed group comprised of 30 participants were not regularly exposed to biotoxins. This was established in an interview and by the modified Q16 neurotoxic symptoms questionnaire in which the level of neurotoxicity would be expected to be less than 32 (i.e. less than low level). Control participants had the same educational level as exposed subjects, which was also established in the interview.

To ensure accuracy, participants must meet specific requirements, including having at least one eye with a corrected visual acuity of 20/50 or better, using a properly functioning device with sufficient pixel density, and testing under adequate lighting conditions (>70 foot-lamberts).

The exclusion criteria for both groups were participants who, at the time of visual assessment, had systemic infections, cancer, systemic and neurological disorders unrelated to environmental toxins, or were pregnant. To ensure compliance with these exclusion criteria, all participants’ eligibility was confirmed through a referral by the General Practitioner (GP) and by the information gathered from the demographic questionnaire. This allowed verification of participants’ health status and ensured that they met the necessary inclusion and exclusion criteria.

At the beginning of testing, a comprehensive visual assessment was performed, and the modified Q16 neurotoxic symptoms questionnaire was administered. Additionally, each participant completed a demographic information questionnaire. The Bailey-Lovie Log MAR chart was used to assess distance visual acuity, and a standardised Bailey-Lovie Near reading chart was used to evaluate near visual acuity at a controlled distance of 40 cm. To ensure measurement accuracy, examiners verified the working distance before each trial, as even small deviations (e.g. an 8 cm reduction) would alter the angular size of the optotypes and artificially influence the recorded acuity. Biomicroscopy (Topcon) was used to assess the presence or absence of lens opacities using the lens opacities classification system III (LOCS III) [19]. Direct ophthalmoscopy and retinal assessment using a 90-diopter lens in combination with a slit lamp biomicroscope were performed to evaluate retinal and overall ocular health. The participants were refracted using an autorefractor (Topcon KR-800), and those who needed refractive correction were corrected with untinted lenses.

For both the exposed and non-exposed groups, all visual tests and questionnaires were conducted at the Optometry Clinic Research examination rooms at the University of New South Wales (UNSW), and VCS testing was conducted in the Sensory Processing Lab at UNSW School of Optometry and Vision Science. The VCS tests were performed under low dim lighting conditions (∼300-500 lux standard for optometric assessment), with regulated temperature and minimal environmental noise to ensure consistency, all on the same day. Between tests, participants were given a rest period of 5 to 7 min to minimise fatigue. This study was conducted in accordance with the ethical principles outlined in the Declaration of Helsinki. Ethical approval was obtained from the Macquarie University Human Research Ethics Committee (Reference: 520221187240023). Written informed consent was obtained from all participants before their inclusion in the study.

### 2.2. Measures of neurotoxicity exposure

#### 2.2.1. The modified Q16 neurotoxic symptoms questionnaire

The Q16 questionnaire, validated in English, is widely utilized to monitor neurotoxic symptoms in working populations [20]. The modified version comprises 16 questions related to neurotoxic symptoms, with responses graded on a five-point Likert scale (strongly disagree, disagree, neutral, agree, strongly agree). To ensure participants fully understood the questions and had the opportunity to clarify any doubts, the questionnaire was administered as an interview by the researchers. The modified version’s scoring ranges are as follows: 6 to 32 (Low), 33 to 48 (Medium-Low), 49 to 64 (Medium), and 65 to 80 (High) [18,19].

#### 2.2.2. Pupil size measurements

Pupil size was measured in a consulting room under standard ambient lighting conditions, with illumination levels maintained at approximately 300–500 lux, consistent with typical examination room lighting. This level of brightness is sufficient for routine clinical assessment without causing significant pupillary constriction or dilation.

A millimetre (mm) ruler was used to directly measure the horizontal diameter of the pupil in both eyes. The patient was instructed to fixate on a distant target to minimize the effects of accommodation. Measurements were taken three (3) times per eye to improve reliability and minimize observer error. Each measurement was recorded in millimetres, and the average of the three readings was calculated and documented as the final pupil size for each eye. This is a standard clinical approach for measuring pupil size in optometric practice [21].

### 2.3. Contrast sensitivity tests

The four VCS tests were performed in a randomized order to minimize potential order effects and reduce bias related to learning, fatigue, or adaptation. Each test was performed once, with a rest interval of 5 to 10 min between assessments. If a participant did not fully understand a test procedure, a short demonstration of the test was given until the test requirements were understood. All tests were conducted under photopic conditions, with the laboratory environment maintained at a standard luminance level of approximately 300–500 lux, which is appropriate for screen-based contrast sensitivity testing. Testing took place in the Sensory Processes Laboratory at UNSW Sydney, under controlled and consistent lighting conditions.

#### 2.3.1. Shoemaker handheld chart

**Figure 1.**
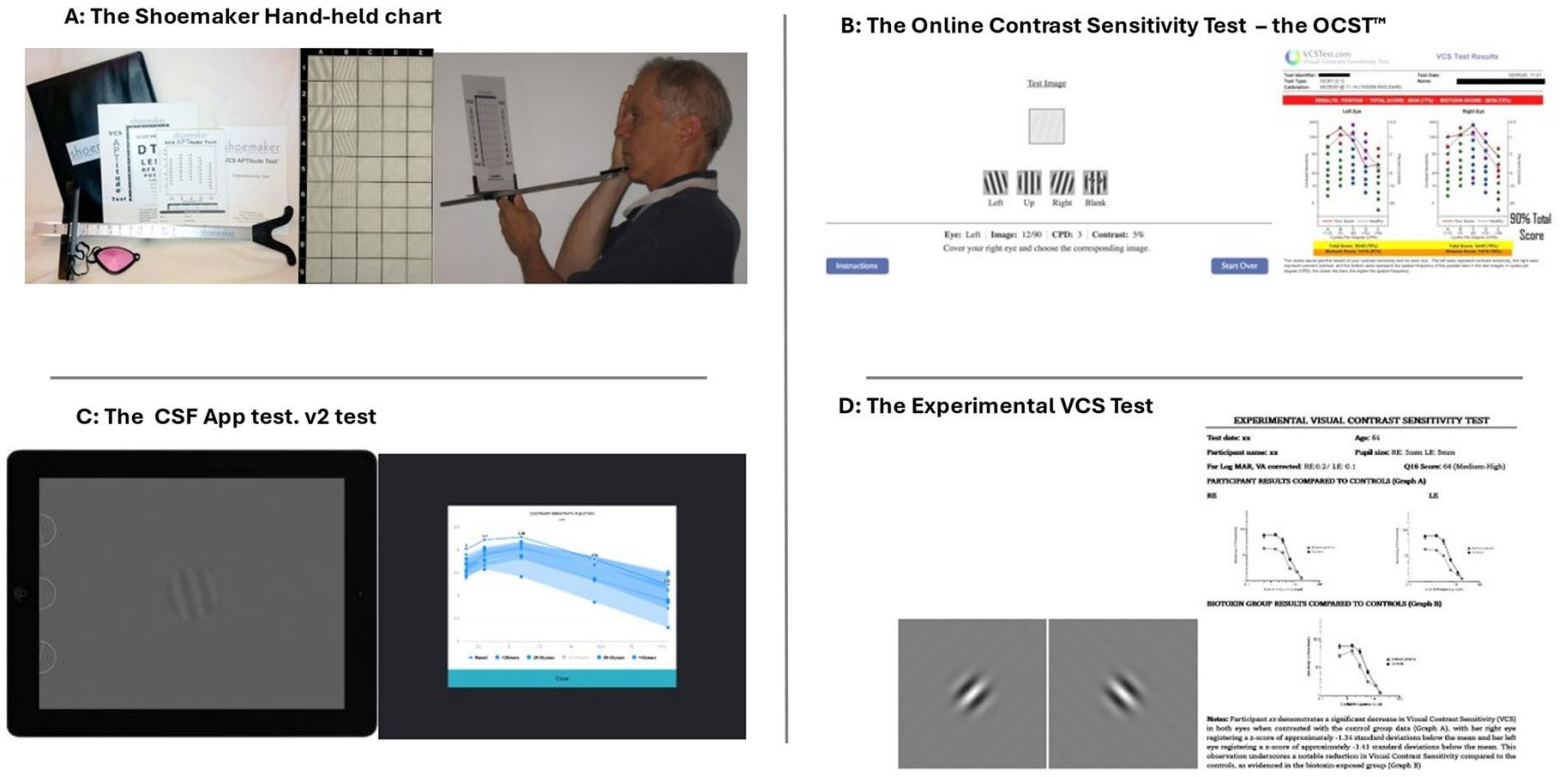
Appearance of the four VCS tests and recording results. (A) Shoemaker hand-held chart; (B) Online Contrast Sensitivity Test – the OCST^TM^; (C) Clinic CSF App test. v2; (D) Experimental VCS.

The VCS test (see Figure 1A) is based on the Functional Acuity Contrast Test (F.A.C.T.) chart for near vision. This tool assesses the ability to detect contrast, which can be impaired by exposure to biotoxins. The test uses sinusoidal gratings and alternating light and dark bars to measure contrast sensitivity across multiple spatial frequencies, typically including 1.5, 3, 6, 12, and 18 cycles per degree (cpd). The test chart consists of rows of gratings with varying contrast levels, decreasing from left to right within each row. During the test, subjects identify the orientation of the gratings (vertical(up), tilted left (left), tilted right (right)), and the lowest contrast level correctly identified for each spatial frequency is recorded using a scoring sheet. Results were compared to normative data to determine if the patient’s contrast sensitivity is within the test’s normal range [22–25], and a contrast sensitivity curve can be plotted to visualize performance across frequencies. Accurate calibration and equal illumination of the VCS chart (70-foot lamberts, normal daytime indoor light) are crucial for obtaining reliable results. The test was performed at 40–45 cm with proper lens correction to achieve a visual acuity of at least 20/50 (0.4 Log MAR). The test was conducted monocularly, starting with the right eye, followed by the left. Subjects select the orientation of the patch from three options: left, up, and right, and the correctness of their response is recorded.

#### 2.3.2. Online contrast sensitivity test (OCST^TM^)

The digital online version of the VCS test (Figure 1B), available through the VCSTest.com platform, provides an accurate and convenient method for assessing contrast sensitivity online. This is also based on the F.A.C.T chart for near vision. Significantly, this test has served as an initial screening tool for CIRS and other biotoxin-related conditions, with results that can be shared with healthcare providers for further evaluation. This test consists of two series of 5 sets of 9 images of sinusoidal grating patterns, administered separately to each eye. Each image varied in spatial frequency and contrast, becoming progressively more challenging to distinguish as the contrast decreased and the bars became narrower (high spatial frequency). Participants were instructed to cover one eye at a time and identify the orientation of the grating (‘Left’, ‘Up’, or ‘Right’) in a forced choice paradigm. An ‘unsure’ option is also available, as such responses provide an indication of the contrast level at which the participant is uncertain about the orientation of the grating stimulus. It is not treated as a correct or incorrect response. Selecting ‘unsure’ does not terminate the test; participants continue until all items are completed.

The psychophysical procedure follows a fixed-step, method-of-limits approach. For each spatial frequency, the contrast of the gratings was decreased in discrete steps, and the participant was required to identify the orientation of the grating. The contrast level at which the participant was not able to correctly identify the grating provided an estimate of the contrast threshold. The contrast sensitivity was calculated as the reciprocal of the lowest contrast level detected for each frequency band, and the CSF was constructed by plotting contrast sensitivity as a function of spatial frequency. No feedback was given to indicate the correctness of the response.

Although the test does not use a fully adaptive staircase method as in more developed psychophysical platforms (e.g. ClinicCSF.v2), its linear decrement protocol and forced-choice response structure ensure consistent and reproducible threshold estimation.

Display calibration using a ruler or standard banking card is strongly recommended. The test dynamically generates images in software, ensuring high quality and variability across administrations. Results follow the same biotoxin-illness pass/fail algorithms proposed by Shoemaker and provide expanded interpretations based on contrast sensitivity-related research [26].

#### 2.3.3. Clinic CSF app test 2.0.1 2022

ClinicCSF.v2 (Figure 1C) was used to measure contrast sensitivity at spatial frequencies of 1.5, 3, 6, 12, and 18 cpd. These were generated using MATLAB and calibrated using a Spyder4Elite colorimeter to ensure accurate luminance and contrast values. The app employs Bit Stealing technology for high-resolution luminance control, offering up to 2,540 luminance levels. Stimuli feature smooth edges to minimize visual artifacts and are presented at a configurable viewing distance [27].

ClinicCSF.v2 utilises a three-step adaptive psychophysical method based on the FACT test. The CS threshold was determined by averaging the contrast levels corresponding to the reversal points (i.e. the contrast levels where the direction of response changed) for five reversals. Gratings are randomly oriented (vertical, 15° left, or 15° right), and contrast sensitivity thresholds are determined based on participant responses. No feedback was given to indicate the correctness of the response.

#### 2.3.4. Experimental VCS test

The Experimental VCS test was a computerized custom software written and conducted in MATLAB (version 11). Participants were presented with an oriented Gabor patch (diameter of 4°of the visual angle) displayed on a linearised iPad screen that was attached and screen extension of a Dell Laptop (Inspiron 153530) and had to judge whether the pattern was tilted 45° to the right or left in a two-alternative forced choice design (Figure 1D). The background was mid-grey and at a luminance of 55 cd/m^2^. The Weber contrast of the stimulus, coinciding with the amplitude of the Gabor stimulus, was modified using a staircase procedure corresponding to the 79.0% correct performance level. Initially, the starting Webber contrast of the stimulus was 0.8, and the step size was 0.08. After the first and subsequent reversals, the step size was halved. After the third reversal, the step size was 0.01 and remained at this value until the end of the staircase trial. The staircase lasted six reversals, and the average of the last four reversals was averaged to estimate the contrast detection threshold. No feedback was given to indicate the correctness of the response. The test was conducted monocularly, and the stimulus presentation was 1 s. The staircase procedure was repeated for the following spatial frequencies: 1.5, 3, 6, 12, and 18 c.p.d., and each observer was measured once at each spatial frequency in a randomized order.

### 2.4. Statistical analysis

Descriptive analyses, including participant demographics, including age and gender, were reported. Data distribution was assessed for normality before analysis. Depending on the results of normality testing, either an independent samples t-test or a Mann-Whitney U test was used to determine statistical significance in the bivariate analyses. Multivariate analyses were performed using a two-way ANOVA. In all cases, a *p* < 0.05 value was considered statistically significant. All analyses were conducted using GraphPad software. The authors confirm that the data supporting the findings of this study are available within the article.

## 3. Results

Participants diagnosed as being exposed to biotoxins exhibited significantly higher modified Q16 neurotoxic symptoms scores compared to non-exposed individuals. Exposed: median 44.5 (IQR 33.0–55.5); Controls: median 22.5 (IQR 19.0–29.0). The group difference was statistically significant (Mann–Whitney *U* = 80.5, *p* < 0.0001, two‑tailed), with a very large effect (Cliff’s delta = 0.81). According to Q16 classification criteria, the exposed group demonstrated moderate levels of neurotoxicity, whereas the non‑exposed group showed low levels.

Average pupil sizes for exposed and non-exposed groups were 3.404 mm (SD: 0.782) and 4.101 mm (SD: 0.295), respectively. There was a significant difference in pupil size between exposed and non-exposed groups (Welch’s t-test: t (34.07) = 4.468, *p* < 0.0001), indicating that the exposed group had significantly smaller pupils than the non-exposed group (mean difference: 0.703 mm ± 0.153).

**Figure 2.**
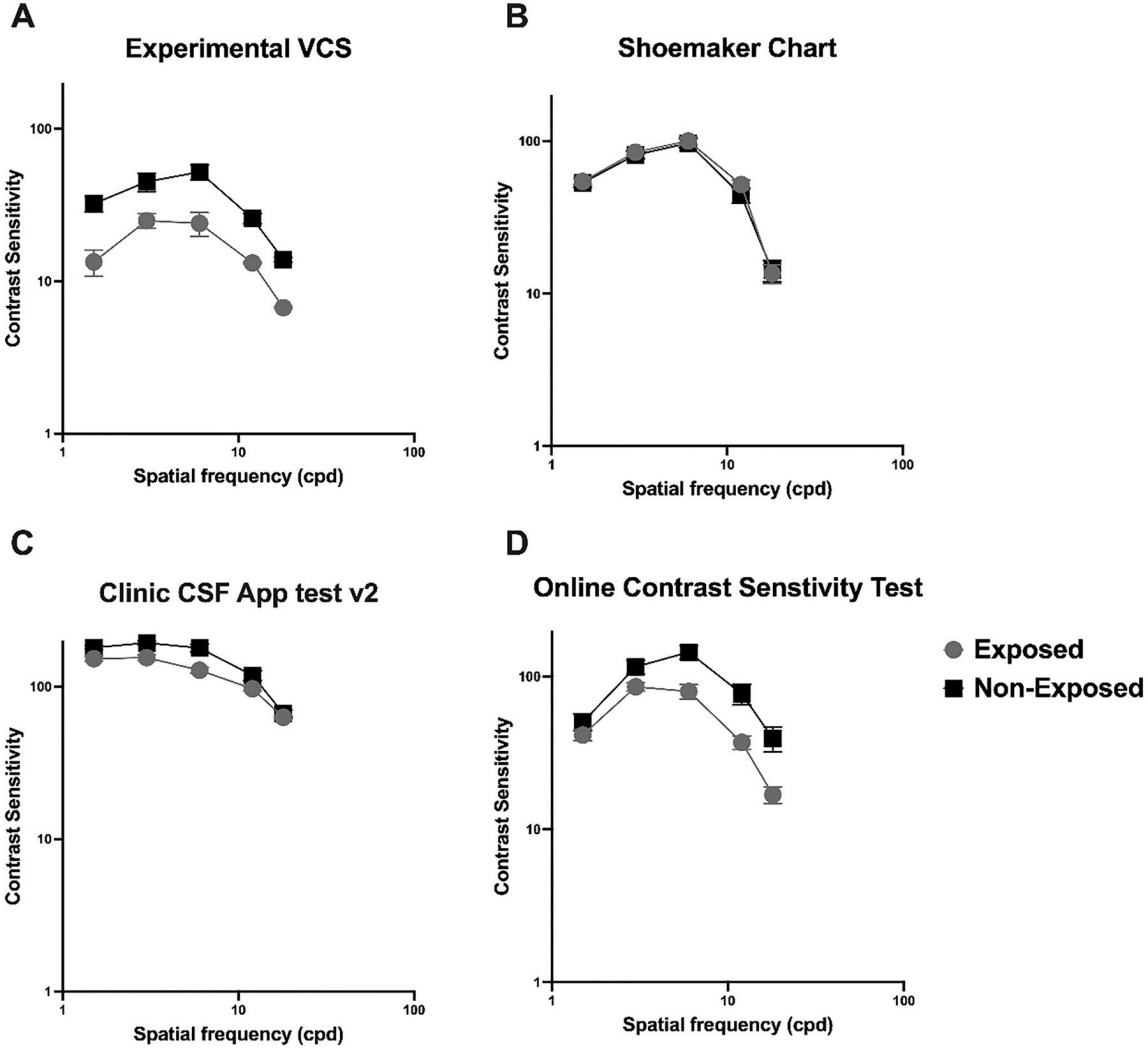
CSFs (contrast sensitivity vs spatial frequency) for exposed and non-exposed groups, shown with different symbols across panels A–D for each of the four VCS tests.

In Figure 2, CSFs (contrast sensitivity vs spatial frequency) for exposed and non-exposed groups (different symbols) are plotted in separate panels (A-D) for each of the four VCS tests used. In all tests, the CSF is lower for the exposed group than the non-exposed group, indicating a reduction in contrast sensitivity. Separate two-way ANOVAs were conducted to determine whether each test was capable of detecting differences in VCS between exposed and non-exposed groups. A summary of the outputs of these analyses is shown in Table 1.

**Table 1.** Two-way ANOVA results for the four different VCS tests examined in the present study.

VCS Test	df (overall)	F	p-value	Partial Eta squared
Shoemaker Chart				
Group	(1,280)	2.561	0.111	0.008
Spatial Frequency	(4, 280)	250.0	<0.0001	0.781
Group x Spatial Frequency	(4, 280)	0.584	0.674	0.000
Online Contrast Sensitivity Test - OCST^TM^				
Group	(1, 280)	99.65	<0.0001	0.267
Spatial Frequency	(4, 280)	94.34	<0.0001	0.574
Group x Spatial Frequency	(4, 280)	7.85	<0.0001	0.101
Clinic CSF App test v2				
Group	(1, 280)	110.8	<0.0001	0.283
Spatial Frequency	(4, 280)	232.6	<0.0001	0.768
Group x Spatial Frequency	(4, 280)	8.552	0.0002	0.109
Experimental VCS				
Group	(1, 280)	138.2	<0.0001	0.406
Spatial Frequency	(4, 280)	47.89	<0.0001	0.3304
Group x Spatial Frequency	(4, 280)	5.75	0.0001	0.076

In general, for all tests, changing spatial frequency significantly affected VCS (see Table 1). As shown in Figure 2, all tests approximated the classic inverted ‘U’ shape (with sensitivity peaking approximately between 4–8 cpd) typical of the CSF. Note that for the Clinic CSF App test. v2, sensitivity values were reported by a factor of 10 to be on the same scale as values reported by the other tests. However, only digital tests showed a significant difference between exposed and non-exposed groups (see Table 1). The Shoemaker handheld chart (though designed to detect signs of biotoxicity) did not show any significant group difference. All three digital tests also showed significant interaction effects, suggesting differences in the shape of the CSF between exposed and non-exposed groups. These results show that whilst all digital tests could detect visual deficits associated with exposure to biotoxins, they do so to different degrees, which may reflect methodological differences and testing procedures. Indeed, partial eta squared calculations showed that the largest group effect size was noted for the Experimental VCS test, followed by the OCST^TM^ and the Clinic CSF App test. v2. Additionally, VCS change as a function of spatial frequency was not consistent for all three tests. Note that the Experimental VCS test showed deficits across the range of spatial frequencies, while the OCST^TM^ at mid to high frequencies, and the Clinic CSF App test. v2 at mid frequencies.

To indicate the reduction in overall contrast sensitivity and a representative of the relative change in the window of visibility, we calculated the area under the curve (AUC) for the exposed and non-exposed groups obtained from the four different tests. To facilitate comparison between the tests, the difference in AUC between exposed and non-exposed was expressed as a proportion of their sum: AUC_diff_ = AUC_exposed_ – AUC_non-exposed_/AUC_exposed_ + AUC_non-exposed_. This calculation showed a relative reduction in AUC for the exposed group by approximately −0.388, −0.281, −0.077, and −0.001 for the Experimental VCS, the OCST^TM^, and Clinic CSF App test. v2, and the Shoemaker handheld chart, respectively. The largest difference was observed for the Experimental VCS test.

The sensitivity and specificity of each of the four tests were derived from the AUC calculations. These values are reported as ranges because each test was evaluated across several contrast‑sensitivity (CS) cut‑off levels rather than a single threshold, and diagnostic accuracy varied accordingly. For the Experimental VCS test, sensitivity ranged from 85% to 100%, while specificity ranged from 60% to 80%, producing a maximum likelihood ratio (LR^+^) of 4.0. Reporting the full range avoids the incompleteness of stating only ‘up to 100%’ and reflects the variation observed across thresholds. A reduction in contrast sensitivity was defined using a moderate‑to‑low CS cut‑off of 1.5 log units, a level commonly associated with functional impairment. In comparison, both the OCST^™^ and the CSF App v2 tests demonstrated high sensitivity (ranging from 80% to 95%) but lower specificity (ranging from 20% to 40%), resulting in LR^+^ values between 1.25 and 2.5, indicating more modest discriminative ability. The Shoemaker handheld chart exhibited the smallest AUC difference, suggesting limited sensitivity to exposure‑related changes

Correlation analyses (Pearson) (see Table 2) were conducted to determine if there was a significant association between the VCS tests and the modified Q16 neurotoxic symptoms questionnaire score for exposed and non-exposed groups. It was hypothesised that higher symptom scores may be negatively associated with AUC values (indicating poorer VCS). The modified Q16 neurotoxic symptoms questionnaire scores were only significantly correlated with AUC values obtained from the Experimental VCS test (r = −0.447, *p* = 0.017), and not with the other tests (Ps > 0.126), for the exposed group only. Additionally, no VCS test was significantly associated with pupil size for both groups (Ps > 0.262).

**Table 2.** Correlations between Visual Contrast Sensitivity (VCS) test results, area under the curve (AUC), and the modified Q16 neurotoxic symptoms questionnaire in exposed and non‑exposed groups, as well as correlations between pupil size and AUC in exposed and non‑exposed groups.

AUC and Modified Q16 neurotoxic symptoms questionnaire	Non-exposed group (Controls)		Exposed group (Cases)	
VCS test	*r*	*p*	*r*	*p*
Shoemaker Handheld Chart	0.290	0.118	−0.297	0.123
Clinic CSF App test 2.0.1 2022	0.195	0.299	−0.076	0.697
Online Contrast Sensitivity Test OCST^TM^	−0.009	0.985	−0.176	0.369
Experimental VCS	0.156	0.409	−0.447	**0.017** *
AUC and Pupil sizes				
VCS test				
Shoemaker Handheld Chart	0.115	0.542	−0.290	0.133
Clinic CSF App test 2.0.1 2022	0.155	0.411	0.024	0.902
Online Contrast Sensitivity Test OCST^TM^	0.141	0.454	−0.174	0.375
Experimental VCS	−0.125	0.507	0.033	0.863

* Significant alpha = 0.05.

## 4. Discussion

In the present study, the capability of four (4) different VCS tests in detecting alterations in vision associated with exposure to biotoxins, particularly mould exposure, was examined. In addition, signs of neurotoxicity were assessed using the modified Q16 and pupil size measurements. The findings demonstrated that the exposed group reported significantly higher neurotoxic symptoms scores and exhibited smaller pupil sizes, indicating possible functional and neural changes associated with biotoxicity. Commercial digital VCS platforms such as the Clinic CSF App and the OCST^TM^ show higher sensitivity but lower specificity in detecting VCS deficits among exposed participants. In contrast, the Experimental VCS test achieved a sensitivity of up to 100% and a specificity ranging from 60% to 80%, suggesting a potentially valuable diagnostic capability. Notably, the significant association between Q16 scores and contrast sensitivity (for the more sensitive Experimental VCS test) also indicates that the modified Q16 questionnaire, despite being a simple, low‑burden screening tool, may itself be sufficient to identify individuals at risk of contrast sensitivity impairment.

Importantly, this finding provides further evidence and validation that support the utility of VCS testing in identifying visual impairments associated with biotoxin exposure, particularly mould. These findings also contribute to the broader literature on the neurological impacts of environmental toxins. Additional studies have similarly documented CS impairment and neurocognitive changes in individuals exposed to damp indoor environments, mould-related mycotoxins, and other bioaerosols, supporting the consistency of these findings [2,28–31]. Although age‑matching was implemented during recruitment to minimise age‑related confounding in contrast sensitivity, other unmeasured demographic or environmental factors may still have contributed to group differences. This should be considered when interpreting the observed effects.

The chart version of the Shoemaker test (despite being recommended as a tool to assess for exposure to biotoxicity) did not show any difference in VCS between exposed and non-exposed groups. While originally developed to offer a rapid approach to the assessment of contrast sensitivity function [2] its limited contrast resolution, fixed spatial frequencies, and susceptibility to lighting and learning effects may reduce its efficacy in subtle deficit detection, especially in settings where rigorous standardisation cannot be guaranteed. This observation aligns with previous analysis regarding the limited diagnostic specificity of fixed-format VCS tests in occupational and environmental neurotoxicology [3].

Other comparative studies have likewise shown improved accuracy when digital psychophysical procedures are used, particularly those employing adaptive staircases and stimulus randomisation [32,33]. Further to previous comments, digital versions of VCS tests were capable of detecting group differences in VCS, as they may offer greater sensitivity in detecting vision change. This may stem from the fact that they are more customisable, allowing for precise and controlled stimulus presentations (typically on calibrated screens) and stimulus randomisation procedures in which stimulus presentations can be repeated/retested (and avoiding learning the stimulus order). Additionally, the adoption of software-controlled and customisable thresholding processes (that incorporate well-established psychophysical staircase procedures and adaptive contrast steps in response to detection judgements) has been well known to improve the accuracy of threshold estimation.

Despite all digital VCS testing showing good sensitivity and specificity in discriminating between exposed and non-exposed groups, the tests do so to differing degrees, and with all tests reporting different spatial frequency-dependent effects. Among the four VCS tests evaluated, the Experimental VCS test had the highest diagnostic performance, evidenced by its strong group effect size, area under the curve (AUC) difference, and specificity/sensitivity profile. This test, which utilised custom MATLAB-based psychophysics with adaptive staircases and finer contrast steps, demonstrated a capacity to detect subtle deficits not captured by the Shoemaker handheld chart or even the OCST^TM^ test. These findings align with prior research indicating that test sensitivity in contrast detection can be markedly influenced by task design, contrast resolution, and spatial frequency sampling density [6,7].

Interestingly, both the Clinic CSF App test v2 and the OCST^TM^ test showed intermediate performance, successfully differentiating the groups but with lower specificity than the Experimental VCS test. The lower specificity could result from the use of broader contrast steps and fixed thresholding algorithms, which, while facilitating efficiency, might decrease sensitivity to subtle gradations in function [4,13].

The differences in the performance of all digital VCS tests most likely stem from the different methodologies, stimuli, testing procedures, and purpose unique to each approach. The Experimental VCS test was specifically developed for detailed psychophysical assessment, where highly precise estimates of contrast sensitivity are required to detect subtle visual changes. Its design relies on an adaptive staircase thresholding procedure, which systematically adjusts contrast in increasingly finer steps to converge on an accurate sensitivity threshold. This methodological approach aligns with well‑established evidence showing that adaptive psychophysical procedures with small contrast increments provide more reliable and precise threshold estimates than fixed‑step methods [34,35]. However, this test can be time consuming as typically the number of presentation trials would be higher to estimate the threshold, which in addition to the fact that it is not commercially available and requires specialised equipment and technical support may mean that it may not be entirely suitable for clinical screening where time efficiency is required. On the other hand, the OCST^TM^ and the CSF App test. v2 test have been designed with visual screening in mind, trading off sensitivity for time efficiency, which was demonstrated in the present study.

All three digital tests offer solutions to detect visual change associated with exposure to biotoxins, depending on the intended purpose. Due to their ease of use and efficiency, both the OCST^TM^ and the CSF App test. v2 tests may be used to quickly screen for VCS deficits. This is particularly useful in a clinical setting where the purpose is to quickly screen for signs of biotoxicity. When comprehensive testing or monitoring is required, particularly to establish the extent of visual change and specifically examining for deficits at different spatial frequencies, Experimental VCS testing may be appropriate.

In addition to VCS, this study supports the use of the modified Q16 neurotoxic symptoms questionnaire as a valid correlate of visual impairment in biotoxin-exposed individuals. Higher Q16 neurotoxic symptom scores in the exposed group were associated with reduced contrast sensitivity (for the more sensitive Experimental VCS), confirming earlier findings linking self-reported neurotoxic symptoms to functional impairments in the visual system [18,20]. This consistency highlights the value of an integrated screening approach, combining objective (VCS) and subjective (modified Q16 neurotoxic symptoms questionnaire) measures to enhance diagnostic reliability and support effective intervention.

Finally, pupil size measurements further corroborated evidence of neurological dysfunction in the exposed group. The exposed group exhibited significantly smaller pupil sizes compared to controls. This is consistent with previous reports of pupil size change associated with neurotoxicity due to pesticide and organic solvent exposure [14]. This finding may reflect dysregulation of autonomic nervous system function, neuroinflammation, or central nervous system change, such as disruption of midbrain structures involved in pupillary control (i.e. the Edinger–Westphal nucleus) [36]. Elevated pro-inflammatory cytokines following mould or mycotoxin exposure can produce neuroinflammation and autonomic dysfunction, providing a possible neurobiological basis [30,31,37]. Reduced pupil size, as a physiological marker, complements the observed VCS impairments and elevated modified Q16 neurotoxic symptoms questionnaire scores, providing additional evidence of biotoxin-associated neural dysfunction involving both sensory processing and autonomic regulation. Appropriate future studies may use more precise instrumentation to quantify pupil sizes to associate this measure with cytokine levels to provide further evidence of biotoxin-related neurological change. Further, constriction of pupil sizes is associated with fatigue [38,39], and higher rates of fatigue have been reported in individuals exposed to biotoxins [16]. Such an investigation is beyond the scope of the present study; this association should be explored.

## 5. Conclusion

This study shows that computerized VCS testing can effectively detect visual deficits in patients with mould-related biotoxins. With increasing awareness of conditions like the CIRS, using sensitive VCS tools in diagnosis could improve early detection, monitoring, and treatment. Future work should aim to optimize their usability in general practice settings and explore the neurological brain changes with neuroimaging.

## Data Availability

The data supporting the findings of this study are available from the corresponding author upon reasonable request. Data sharing is subject to ethical and privacy considerations.
